# Moloney murine leukemia virus glyco-gag facilitates xenotropic murine leukemia virus-related virus replication through human APOBEC3-independent mechanisms

**DOI:** 10.1186/1742-4690-9-58

**Published:** 2012-07-24

**Authors:** Takayuki Nitta, Sangouk Lee, Dat Ha, Maribel Arias, Christine A Kozak, Hung Fan

**Affiliations:** 1 Department of Molecular Biology and Biochemistry and Cancer Research Institute, University of California, Irvine, CA, 92697-3905, USA; 2 Laboratory of Molecular Microbiology, National Institute of Allergy and Infectious Diseases, Bethesda, MD, 20892-0460, USA

**Keywords:** glyco-gag, M-MuLV, XMRV, APOBEC3, Virus release, Infectivity, Restrictive factor

## Abstract

**Background:**

One of the unique features of gammaretroviruses is that they contain an additional extended form of Gag, glyco-gag, which initiates in the leader sequence. MuLV glyco-gag, gPr80^Gag^, promotes retrovirus replication and disease progression. Although virtually all infectious MuLVs encode glyco-gag, XMRV (xenotropic murine leukemia virus-related virus) lacks the classical gPr80^Gag^ sequence. We examined XMRV to determine if its leader sequence contains glyco-gag activity, whether the presence of conventional gPr80^Gag^ affects replication of XMRV, and we describe the evolution of glyco-gag-deficient MuLVs in Mus.

**Results:**

We introduced several mutations disrupting two putative but noncanonical glyco-gag proteins in the leader sequence region in XMRV and found that those mutations did not affect virus release nor susceptibility to the antiviral activity of hA3G (human APOBEC3G). A chimeric XMRV encoding the Moloney MuLV (M-MuLV) leader sequence (MXMRV) demonstrated that M-MuLV glyco-gag facilitated MXMRV release and increased infectivity. Infectivity assays with several cell lines showed that glyco-gag increases XMRV infectivity in all cell lines tested, but the level of this increase varies in different cell lines. Because MuLV glyco-gag counteracts mouse APOBEC3, we investigated whether M-MuLV glyco-gag enhances XMRV infection by counteracting human APOBEC3. Comparison of hAPOBEC3 isoforms expressed in different cell lines indicated that hA3B was the most likely candidate for a restrictive hA3. However over-expression of hA3B showed no enhanced restriction of infection by XMRV compared to MXMRV. Endogenous MuLVs in the sequenced mouse genome were screened for canonical glyco-gag, which was identified in two clades of xenotropic MuLVs (X-MuLVs) and ecotropic MuLVs, but not in other X-MuLVs or in any polytropic MuLVs.

**Conclusions:**

M-MuLV glyco-gag facilitates XMRV replication, and the leader sequence region in XMRV does not encode proteins equivalent to M-MuLV glyco-gag. The fact that the ability of glyco-gag to enhance XMRV infection varies in different cell lines suggests a glyco-gag sensitive restrictive factor that further reduces XMRV infectivity. The M-MuLV glyco-gag enhancement for XMRV replication is through a hAPOBEC3 independent mechanism. The absence of glyco-gag in MuLVs carried by western European mice suggests that loss of this sequence is a relatively recent event with limited subspecies distribution.

## Background

Gammaretroviruses infect a variety of host species and are causative agents of various diseases including malignant tumors, neurological disorders and immunodeficiency [1-4]. One of the unique features of gammaretroviruses is that many encode an additional form of Gag protein, glycosylated Gag (glyco-gag). In Moloney murine leukemia virus (M-MuLV) glyco-gag (gPr80^*gag*^) is translated from unspliced viral mRNA via an upstream CUG initiation codon in frame with the AUG initiation codon for the Pr65^*gag*^ polyprotein precursor for the viral core proteins [5-7]. gPr80^*gag*^ contains 88 additional amino-terminal (N-terminal) amino acids, including a signal peptide that leads to transport of the protein into the rough endoplasmic reticulum, where it is glycosylated and exported to the cell surface [8]. At the cell surface, mature gPr80^*gag*^ is cleaved into two proteins of 55 (N-terminal) and 40 (C-terminal) kDa. The 55 kDa portion is maintained in a type II integral membrane configuration, with the unique 88 amino acids in the cytosol [5,8,9]. In mice, gPr80^*gag*^ is a major pathogenic determinant for neuropathic FrCasE MuLV [10-12]. MuLVs mutant in gPr80^*gag*^ show replication defects in mice, and there is a strong selection for recovery of glyco-gag function [13-15]. Recently we found that glyco-gag facilitates viral assembly or release through an interferon-sensitive pathway, and in particular through lipid rafts [15-17]. Other investigators have recently reported that gPr80^*gag*^ can complement a replication defect for Nef-negative HIV-1 [18], and that gPr80^*gag*^ antagonizes restriction of MuLV by mouse APOBEC3 (mA3, apolopprotein B mRNA-editing enzyme catalytic polypeptide 3) both in vitro and in vivo [19].

Recently a novel infectious gammaretrovirus related to MuLVs has been discovered that can infect human cells [20,21]. This virus, xenotropic murine leukemia virus-related virus (XMRV), shares 94% overall sequence similarity with xenotropic and polytropic endogenous MuLV proviruses in the mouse genome. Xenotropic MuLVs cannot infect laboratory mouse strains because of lack of a functional receptor, but they can infect cells of wild mouse species and other species including humans [22,23]. XMRV infection was initially associated with human prostate cancer and chronic fatigue syndrome, but these associations have generally been refuted [24-26]. Very recently it has been shown that XMRV arose by recombination between two specific endogenous MuLV proviruses (preXMRV-1 and preXMRV-2) during in vivo passage of a human prostate cancer xenograft in nude mice [26]. Nevertheless, because it is infectious, XMRV provides a useful tool to study the biology of endogenous xenotropic MuLVs which were presumably infectious in progenitors of modern laboratory mice when they endogenized [23].

Although XMRV is no longer suspected to be a human pathogen, it is an infectious virus with sequence and phenotypic differences relative to mouse-derived MuLVs. Similar to M-MuLV and other exogenous gammaretroviruses, XMRV has a leader sequence in the 5’ end of the viral RNA genome upstream of the AUG for Pr65^*gag*^. However, unlike virtually all infectious MuLVs, XMRV has no contiguous open reading frame upstream of the Pr65^*gag*^ AUG, so it cannot encode a classical gPr80^*gag*^-like glyco-gag [20,27]. On the other hand, this region does contain two CUG codons followed by in frame coding sequences that could potentially encode small proteins that might have glyco-gag function. In this report we tested this possibility and found no evidence for glyco-gag activity in the leader sequence region of XMRV. We also generated a chimeric virus expressing M-MuLV glyco-gag in frame with XMRV Gag, MXMRV. Studies with MXMRV revealed a restriction factor for XMRV in some but not all human cell lines tested that could be counteracted by M-MuLV glyco-gag, but was independent of human APOBEC3 expression. We also determined that, while virtually all infectious MuLVs have an apparently functional glyco-gag sequence, XMRV and some subsets of endogenous MuLVs lack glyco-gag, suggesting that the infectious MuLV progenitors of these proviruses were glyco-gag free.

## Results

### XMRV does not encode glyco-gag activity

**Figure 1:**
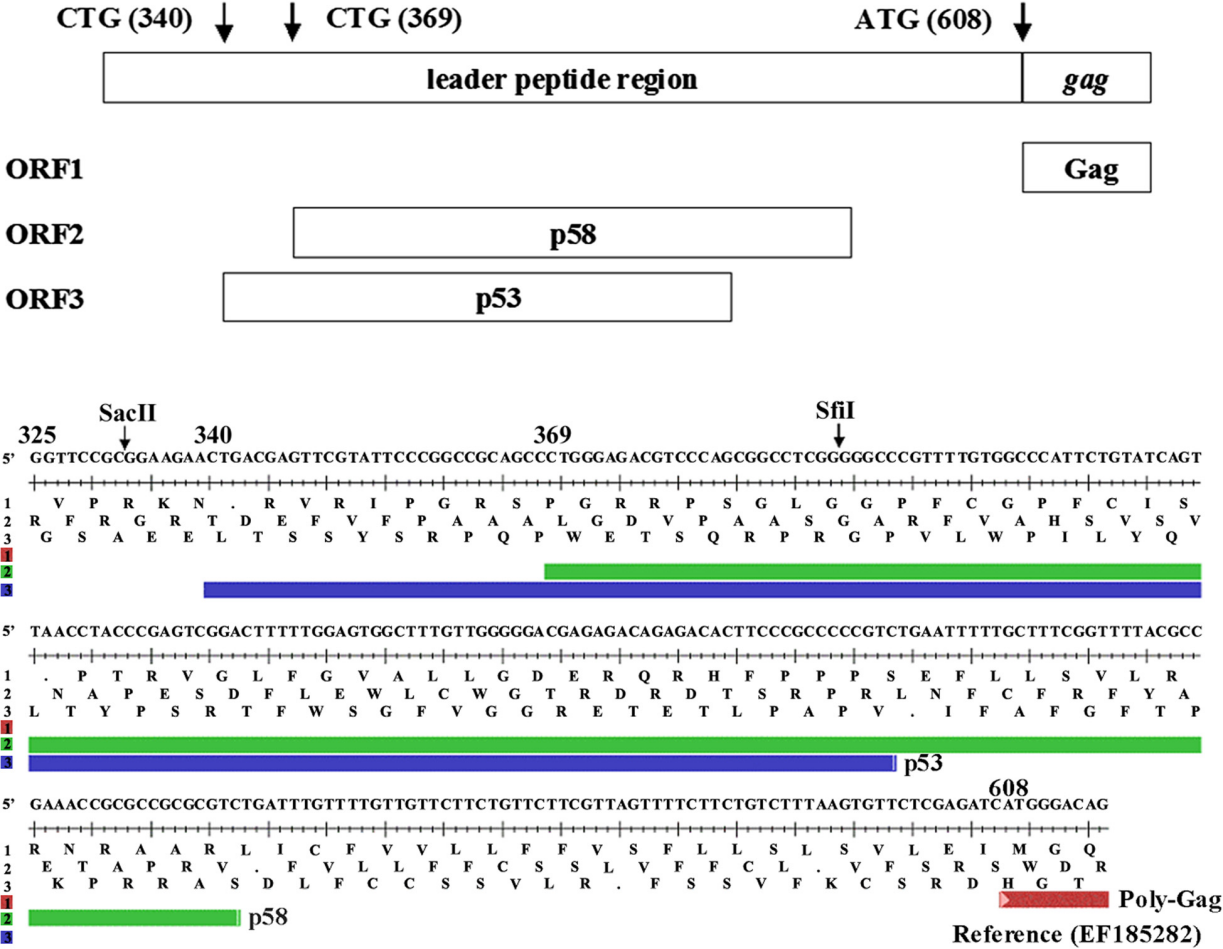
**Potential glyco-gag proteins in XMRV.** The sequences from the 5’ end of the XMRV genome are shown, with potential open reading frames. The ATG initiation codon for the Gag polyprotein (poly-Gag) is at nt 608. Two potential CTG initiation codons at nt 340 and 369 followed by open reading frames to give the potential proteins p53 and p58 are shown.

Urisman et al.. [20] reported that XMRV has a CUG codon in the 5’ leader sequence 270 bp upstream from the Pr65^*gag*^ AUG initiation codon, analogous to exogenous MuLVs such as Moloney and Friend MuLVs (M-MuLV and F-MuLV). However the leader sequences of XMRV differ relative to M- and F-MuLV, and some endogenous X-MuLVs (see below). XMRV has a 24 bp deletion in the leader sequence, as well as an additional 1 bp deletion, so this CUG would encode a 53 amino acid protein (p53) from a different reading frame than Pr65^*gag*^ (Figure 1). Inspection of the XMRV leader sequence also identified a second CUG in a different reading frame followed by an ORF that could potentially encode a protein of 58 amino acids (p58). Neither of these putative proteins contain the transmembrane domain of the standard glyco-gag sequence. Because glyco-gag in M- and F-MuLV is associated with efficient viral replication [15-19], and the N-terminus contains essential sequences for this activity [17,18], we tested if the putative small proteins in the XMRV leader sequence might have biological functions equivalent to glyco-gag. We introduced mutations that would disrupt expression of p53, p58 (or both) in a plasmid containing the full-length XMRV molecular clone, pVP62 (Table 1).

**Table 1:** The mutations disrupting expression of putative glyco-gag proteins in XMRV

**Constructs**	**mutation**	**XMRV p53**	**XMRV p58**
pVP62 (WT)	None	**+**	**+**
pVP62Δgg0	_ to A (nt 372)	**-**	**-**
pVP62Δgg1	C to AT (nt 382)	**-**	**-**
pVP62Δgg2	T to C (nt 370)	**+**	**-**
pVP62Δgg3	T to C (nt 341)	**-**	**+**
pVP62Δgg4	T to G (nt 354)	**-**	**+**

The mutations to disrupt expression of putative glyco-gag proteins, p53 and p58, in the leader sequence region of XMRV VP62 are shown. Reference for original sequence is EF185282.

We previously showed that gPr80^*gag*^ enhances M-MuLV particle release from infected NIH3T3 cells by directing release through lipid rafts [16]. Compared to glyco-gag mutant virus, wild-type viral particles had higher cholesterol content, reduced buoyant density, and higher sensitivity to the cholesterol depleting agent methyl-beta-cyclodextrin (MβCD) for release [16]. The wild-type pVP62 and the mutants (pVP62Δgg0-4) were transiently transfected into human 293 T cells, and the amounts of intracellular Pr65^*gag*^ protein as well as released virus were assessed by SDS-PAGE and western blotting for M-MuLV capsid (CA, p30^CA^) as described previously [16]. All of the mutant constructs gave similar levels of intracellular Gag protein and released virus. In addition, treatment with MβCD of 293 T cells transfected with pVP62 or the mutant plasmids showed inhibition of virus release, but the MβCD sensitivities were similar for all plasmids (not shown). Thus, there was no evidence that the putative p53 and p58 reading frames upstream of Pr65^*gag*^ were important for XMRV release.

**Figure 2:**
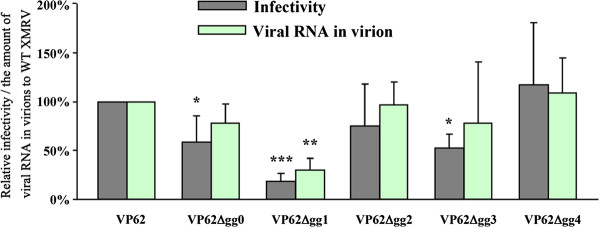
**Infectivity of XMRV mutants.** The wild-type and putative glyco-gag-mutated XMRV stocks (generated by transient transfection of expression vectors in 293 T cells) were used to measure infectivity on HeLa cells by a focal immunofluorescence assay. The amount of viral RNA in virions was determined by quantitative RT-PCR. Both infectivity and the amount of viral RNA were normalized based on Capsid protein in viruses, and the relative infectivities and the amounts of viral RNA relative to wild-type XMRV are shown. Student’s t-test with equal variances was conducted to detect the difference between mean scores in the two groups and values significantly different from wild-type XMRV are indicated (^*^, p < 0.05, ^**^, p < 0.005, ^***^, p < 0.0005).

gPr80^*gag*^ can complement a replication defect for Nef-deficient HIV-1 in human lymphocyte cell lines [18], and it can counteract mouse APOBEC3 (mA3) restriction of MuLV replication in mouse cells [19]. We therefore tested the five mutant XMRVs (obtained by transient transfection of 293 T cells) for infectivity in HeLa cells (Figure 2). The mutant VP62Δgg1 showed a major reduction in infectivity (80%) and 2 mutants (VP62Δgg0 and VP62Δgg2) showed minor reductions (20-40%). Quantitative RT-PCR revealed that these mutants also showed reductions in viral RNA in virions; for these mutants, the reductions in infectivity could have reflected reduced RNA packaging. However the mutants VP62Δgg2 and VP62Δgg4, defective in the putative p58 and p53 respectively, showed no reduction in infectivity or RNA content. Thus neither p58 nor p53 is required for viral infectivity.

We also generated wild-type and mutant XMRVs containing human APOBEC3G (hA3G) that has been shown to restrict XMRV infection [28-31]. 293 T cells were co-transfected with the XMRV expression plasmids along with an expression plasmid for epitope-tagged hA3G. hA3G was incorporated into the wild-type and mutant XMRV virions with equal efficiencies (not shown). XMRV containing hA3G showed an 80% reduction in infectivity in HeLa cells, consistent with previous reports [28,31] (Table 2). The mutant XMRVs showed equivalent reductions in infectivity, indicating that the putative p53 and p58 proteins did not counteract the inhibitory effects of hA3G.

**Table 2:** The mutations for putative glyco-gag did not influence anti-viral activity of hA3G in XMRV infection

**Virus**	**hA3G**	**Trial 1**		**Trial 2**		**Ave ± Std**	
		IU/ml^*^	Relative infectivity^**^	**IU/ml**	**Relative infectivity**	**IU/ml**	**Relative infectivity**
VP62	**-**	1.7 X 10^4^	1	1.4 X 10^4^	1	1.5 X 10^4^ ± 2.3 X 10^3^	0.209 ± 0.029
	**+**	3.2 X 10^3^	0.188	3.2 X 10^3^	0.229	3.2 X 10^3^ ± 3.3 X 10^1^	
VP62Δgg0	**-**	8.4 X 10^3^	1	1.2 X 10^4^	1	1.0 X 10^4^ ± 2.3 X 10^3^	0.196 ± 0.058
	**+**	1.3 X 10^3^	0.155	2.8 X 10^3^	0.237	2.0 X 10^3^ ± 1.1 X 10^3^	
VP62Δgg1	**-**	1.2 X 10^3^	1	3.4 X 10^3^	1	2.3 X 10^3^ ± 1.5 X 10^3^	0.240 ± 0.014
	**+**	3.0 X 10^2^	0.250	7.7 X 10^2^	0.230	5.4 X 10^2^ ± 3.3 X 10^2^	
VP62Δgg2	**-**	1.8 X 10^4^	1	9.1 X 10^3^	1	1.3 X 10^4^ ± 6.0 X 10^3^	0.234 ± 0.010
	**+**	4.0 X 10^3^	0.227	2.2 X 10^3^	0.241	3.1 X 10^3^ ± 1.3 X 10^3^	
VP62Δgg3	**-**	1.1 X 10^4^	1	8.2 X 10^3^	1	9.5 X 10^3^ ± 1.8 X 10^3^	0.214 ± 0.068
	**+**	2.8 X 10^3^	0.262	1.4 X 10^3^	0.165	2.1 X 10^3^ ± 1.0 X 10^3^	
VP62Δgg4	**-**	2.2 X 10^4^	1	1.3 X 10^4^	1	1.7 X 10^4^ ± 6.2 X 10^3^	0.258 ± 0.049
	**+**	6.4 X 10^3^	0.292	2.9 X 10^3^	0.223	4.7 X 10^3^ ± 2.5 X 10^3^	

The 293 T cells were transfected with the XMRV expressing plasmid pVP62 along with the hA3G-V5 or control (pcDNA3) plasmids. The viruses were gathered 2 days post-transfection and were used for infectivity assay with HeLa cells. ^*^The infectious units (IU) were measured from the number of infectious centers that was normalized by the amount of Capsid in viruses and IU per ml was shown. ^**^The relative infectivity to the HeLa cells infected with the viruses released from the 293 T cells transfected with control plasmid was shown.

### M-MuLV glyco-gag facilitates XMRV release through lipid rafts

**Figure 3:**
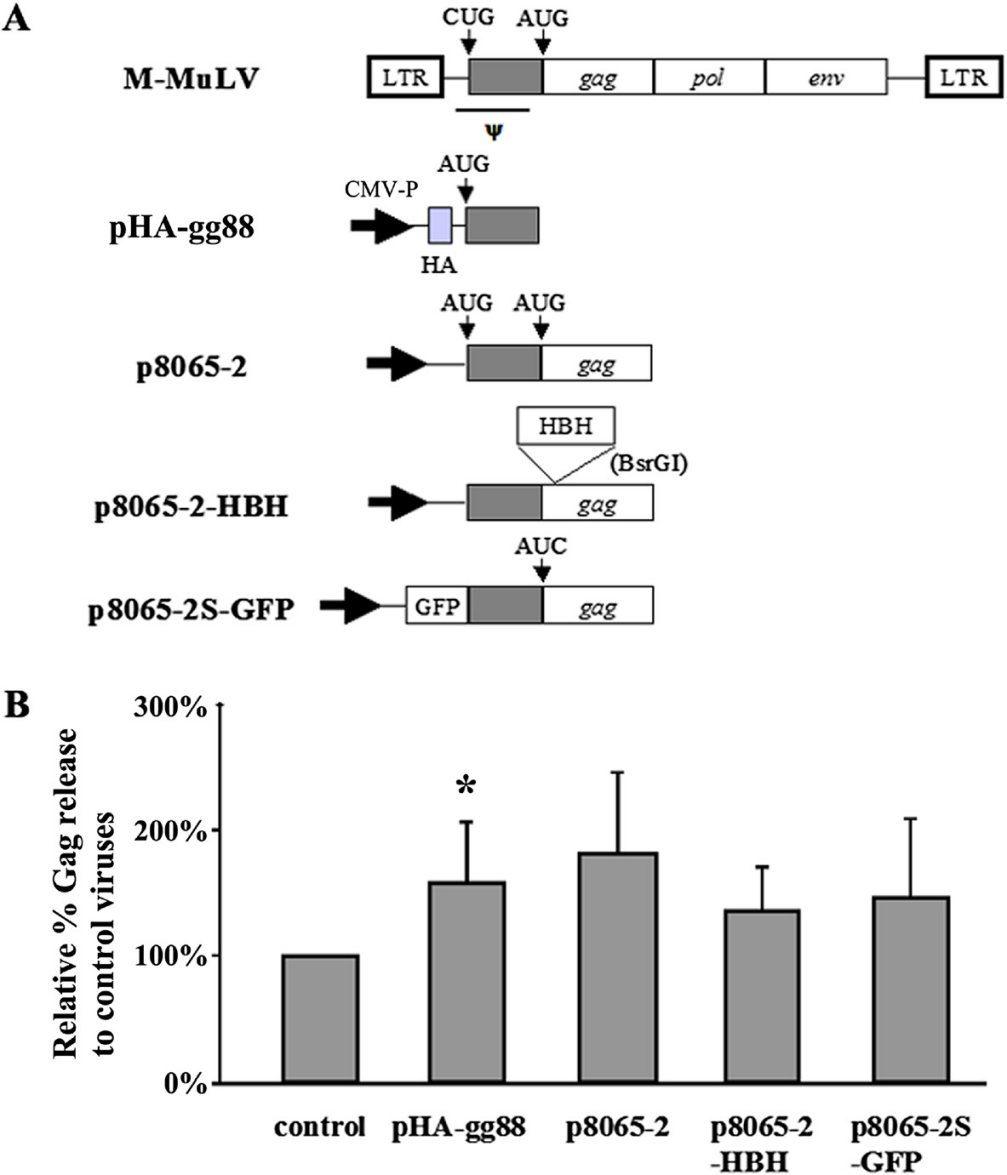
**M-MuLV glyco-gag facilitates XMRV release.** A) The organizations of M-MuLV glyco-gag expression plasmids are shown. In some cases the CTG (CUG) initiation codon for glyco-gag was changed to ATG (AUG) to enhance expression of glyco-gag. B) pVP62 and the glyco-gag expression vectors were co-transfected into 293 T cells and Gag proteins in the released viruses and the cell lysates were detected by SDS-PAGE and Western blots with anti-p30^CA^ antibodies. The amount of viral release relative to control viruses is shown. Welch’s *t*-test with unequal variances was conducted to detect the difference between mean scores in the two groups and values significantly different from pVP62 alone (control) are indicated (*, p < 0.01).

Since XMRV does not appear to encode a functional glyco-gag, we tested if M-MuLV glyco-gag could enhance virus release through lipid rafts. 293 T cells were co-transfected with pVP62 along with expression constructs for different forms of M-MuLV glyco-gag, including full-length protein (p8065-2) and the essential amino-terminal 88 amino acids (pHA-gg88), as well as epitope-tagged proteins (Figure 3 and Additional file 1: Figure S1). The different glyco-gag expression constructs enhanced XMRV release, although to an intermediate level (ca. 50% increase) compared to enhancement of M-MuLV release (2-4-fold, [16,17]).

**Figure 4:**
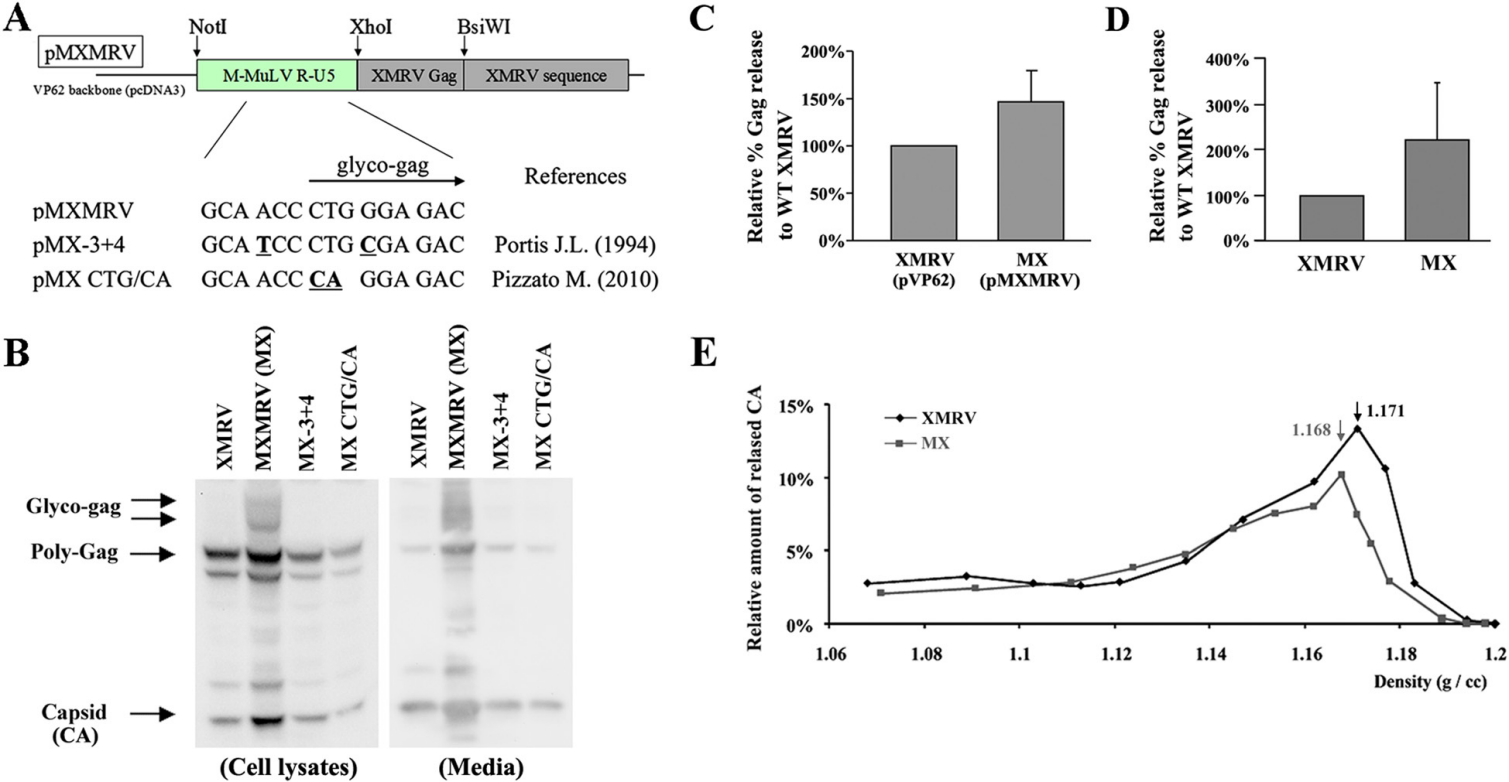
**Comparison of XMRV and MXMRV.** A) Organization of chimeric expression plasmids is shown. pMXMRV encodes M-MuLV glyco-gag fused to XMRV Gag protein. To abolish glyco-gag expression from MXMRV virus (MX), the pMXMRV mutants, pMX-3 + 4 and pMX CTG/CA were generated by substituting nucleotides in or adjacent to the glyco-gag intiation codon (underlined) analogous to glyco-gag negative mutations generated by other investigators (indicated). B) Viruses harvested from 293 T cells transfected with the different XMRV expression plasmids. In addition, released viruses were infected into 293 T cells or DU-145 cells and producer cultures were obtained. In this panel the expression of glyco-gag, poly-Gag and Capsid (CA) proteins in the cell lysates and released viruses from the infected 293 T cells were detected by SDS-PAGE and Western blotting with anti-p30^CA^ antibody. C) Quantification of the relative % Gag release for pMXMRV compared to pVP62 in the transiently transfected 293 T cells is shown. D) Quantification of XMRV and MXMRV released from the infected DU145 cells. E) Buoyant density in 20-55% sucrose gradients of XMRV (diamonds) and MXMRV (squares) released from infected 293 T cells. The densities of each sucrose gradient fraction were measured, and the amount of viral CA protein in each fraction was quantified by SDS-PAGE and Western blotting. Distribution of the percent of total CA in each gradient fraction was calculated, and the results are displayed relative to the densities of each fraction. The peak densities for XMRV and MXMRV are indicated.

We next tested if a chimeric XMRV virus expressing glyco-gag would show enhanced release through lipid rafts. The R, U5 and leader sequences from M-MuLV were substituted into the equivalent region of XMRV pVP62, with the M-MuLV glyco-gag sequences in-frame with XMRV Gag, giving the plasmid pMXMRV (Figure 4A). We also made two mutants in sequences surrounding the glyco-gag CUG initiation codon previously shown to impair expression of glyco-gag [11,18]. pMXMRV and the mutant derivatives were transiently transfected into 293 T cells, and supernatants from the cells were used to infect other 293 T cells. As expected, the cells infected with MXMRV virus showed expression of glyco-gag (the broad band of 85–95 Kd, Figure 4B). Cells infected with MXMRV showed enhanced release of virus as indicated by the amount of Gag proteins. Moreover the MXMRV mutants neither expressed glyco-gag, nor did they show enhanced virus release. Thus in the context of infectious virus, M-MuLV glyco-gag could facilitate release of XMRV, although the enhancement was again intermediate compared to effects on M-MuLV release [16]. This was also true for 293 T cells transiently transfected with pMXMRV vs. pVP62 (Figure 4C and Additional file 2 Figure S2), and for human DU145 prostate cancer cells productively infected with the two viruses (Figure 4D).

Cells infected with MXMRV also showed enhanced (1.5-2-fold) association of Pr65^*gag*^ with detergent-resistant membranes (not shown), and MXMRV virions had a lighter buoyant density than XMRV (Figure 4E). Both of these properties were consistent with enhanced release of MXMRV through lipid rafts.

### M-MuLV glyco-gag enhances XMRV infectivity

**Figure 5:**
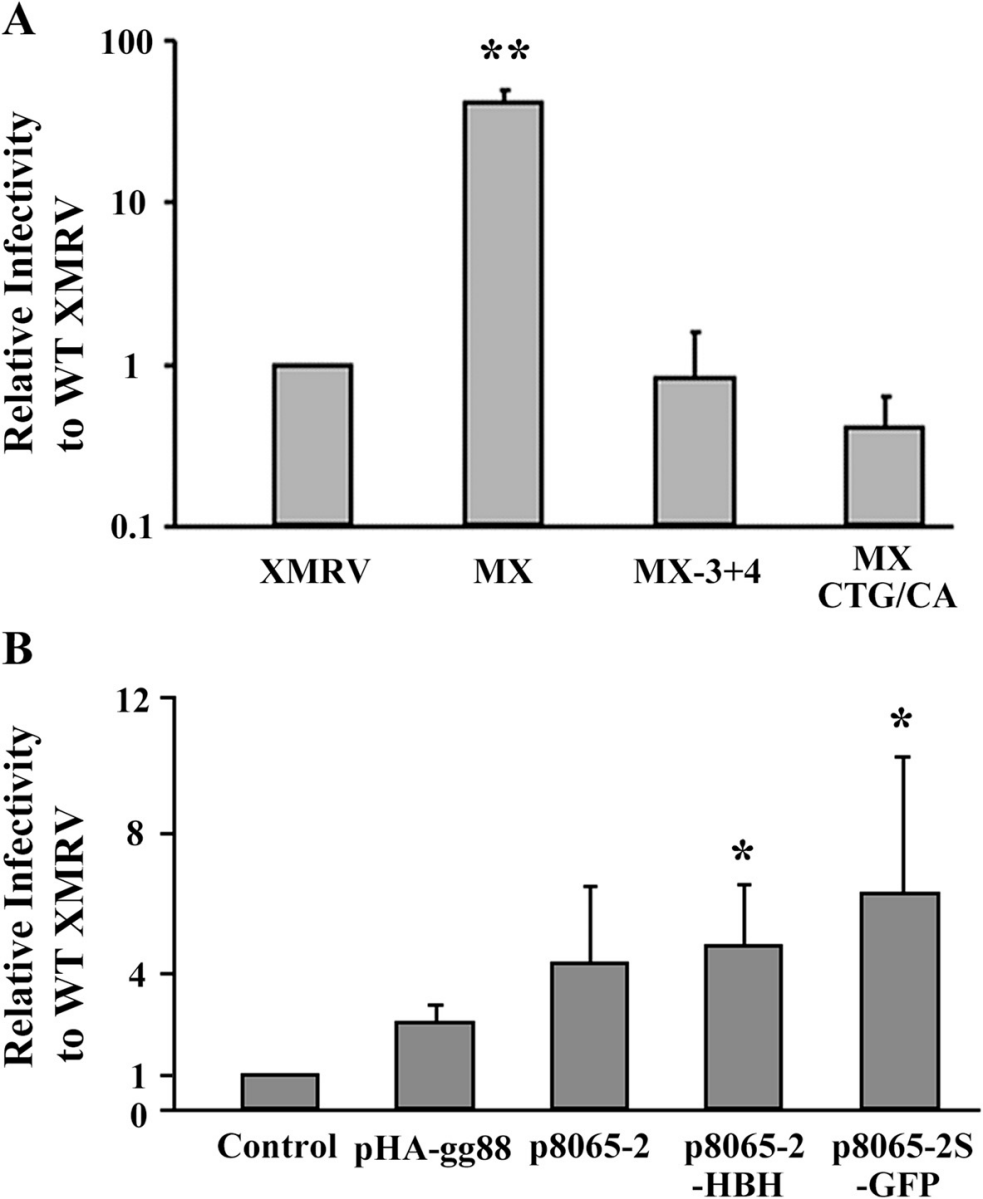
**M-MuLV glyco-gag enhances XMRV infectivity.** A) Viruses were gathered from stably infected 293 T cells and measured for infectivity by the focal immunofluorescence assay (FIA) in HeLa cells. Infectivities were normalized by the amount of Capsid (CA) in each stock and shown relative to wild-type XMRV. B) pVP62 was co-transfected with different M-MuLV glyco-gag expression vectors into 293 T cells and viruses gathered from the supernatants were titered in HeLa cells by FIA. Infectivities were normalized for the amount of CA, and the infectivities relative to control XMRV (co-transfection with empty expression vector) are shown. Welch’s *t*-test with unequal variances was conducted to detect the difference between mean scores in the two groups and values significantly different from XMRV (A) or pXMRV control (B) are indicated (*, p < 0.05. **, p < 0.01).

We next investigated the relative infectivities of MXMRV and XMRV by titration on human HeLa cells, using a focal immunofluorescence assay [15]. As shown in Figure 5A, when equal amounts of the two viruses (normalized by CA content) were assayed, the infectivity of MXMRV was substantially (ca. 36 -fold) higher than that for XMRV. The difference in infectivity reflected glyco-gag since the two MXMRV mutants showed infectivities equivalent to XMRV. Thus M-MuLV glyco-gag can significantly enhance the infectivity of XMRV.

We also tested if glyco-gag could be supplied in trans by transiently co-transfecting 293 T cells with pVP62 and the different glyco-gag expression plasmids, followed by titration of cell supernatants for infectivity in HeLa cells as above (Figure 5B). Co-expression of glyco-gag in XMRV-expressing cells resulted in viruses with enhanced infectivity.

### Cell-specific restriction of XMRV counteracted by glyco-gag

We also tested the relative infectivities of MXMRV and XMRV in several human cell lines as well as rat NRK cells (Table 3). The same viral stocks (normalized for CA protein) from productively infected 293 T cells were titrated on the different cell lines (2–4 titrations). It was interesting that the ratio of MXMRV/XMRV infectivity ranged from 36 in HeLa cells to 2–4 for 293 and HepG2 cells. In fact the titers of the MXMRV stock measured on these three cells were similar (e.g. 3.8 X 10^6^ IU/ml for HeLa vs. 3.9 X 10^6^ IU/ml for 293). However, while XMRV was somewhat less infectious in 293 cells (1.1 X 10^6^ IU/ml) it was substantially less infectious in HeLa cells (1.1 X 10^5^ IU/ml). This indicated a restriction for XMRV in HeLa cells that could be counteracted by M-MuLV glyco-gag; the amount or strength of the restriction was less in 293 cells where the infectivity of XMRV was more similar to MXMRV. DU145 cells also showed a large MXMRV/XMRV infectivity ratio (20.8), consistent with the presence of glyco-gag sensitive restriction. However in these cells the infectivity of MXMRV was lower than for the other human cell lines, suggesting that these cells also may be less infectable in general. NRK cells also showed an MXMRV/XMRV infectivity ratio indicative of glyco-gag sensitive restriction (14.5).

**Table 3:** Comparing cell lines to infection with XMRV and MX viruses

**Recipient cells**	**Virus**	**Trial 1**	**Trial 2**	**Trial 3**	**Trial 4**	**Ave ± Std**		**Ave**
		IU/ml^*^	**IU/ml**	**IU/ml**	**IU/ml**	**IU/ml**	Relative infectivity**	**MX/XMRV**
HeLa	XMRV	1.0 X 10^5^	8.8 X 10^4^	1.6 X 10^5^	8.0 X 10^4^	1.1 ± 0.38 X 10^5^	1 ± 0	36.0
	MX	4.3 X 10^6^	3.1 X 10^6^	4.5 X 10^6^	3.1 X 10^6^	3.8 ± 0.78 X 10^6^	36.0 ± 6.52	
293	XMRV	5.9 X 10^5^	1.2 X 10^6^	1.4 X 10^6^		1.1 ± 0.42 X 10^6^	9.24 ± 3.64	3.89
	MX	3.5 X 10^6^	4.2 X 10^6^	4.2 X 10^6^		3.9 ± 0.38 X 10^6^	35.9 ± 11.1	
HepG2	XMRV		8.7 X 10^5^	2.0 X 10^6^	9.6 X 10^5^	1.3 ± 0.63 X 10^6^	11.4 ± 1.28	2.95
	MX		2.0 X 10^6^	7.7 X 10^6^	2.5 X 10^6^	4.1 ± 3.2 X 10^6^	33.6 ± 12.4	
NRK	XMRV	1.2 X 10^5^	2.9 X 10^4^			7.4 ± 6.3 X 10^4^	0.754 ± 0.593	14.5
	MX	1.2 X 10^6^	8.4 X 10^5^			1.0 ± 0.29 X 10^6^	10.9 ± 2.01	
DU145	XMRV	8.4 X 10^3^	5.1 X 10^3^			6.7 ± 2.3 X 10^3^	0.0705 ± 0.0176	20.8
	MX	1.5 X 10^5^	1.2 X 10^5^			1.4 ± 0.21 X 10^5^	1.47 ± 0.0846	

The several cell lines were infected with XMRV or MX viruses that were produced from the infected 293 T cells (293 T/XMRV and 293 T/MX). *The infectious units (IU) were measured from the number of infectious centers that was normalized by the amount of Capsid in viruses and IU per ml was shown. **The relative infectivity to HeLa cells infected with XMRV viruses was shown.

**Figure 6:**
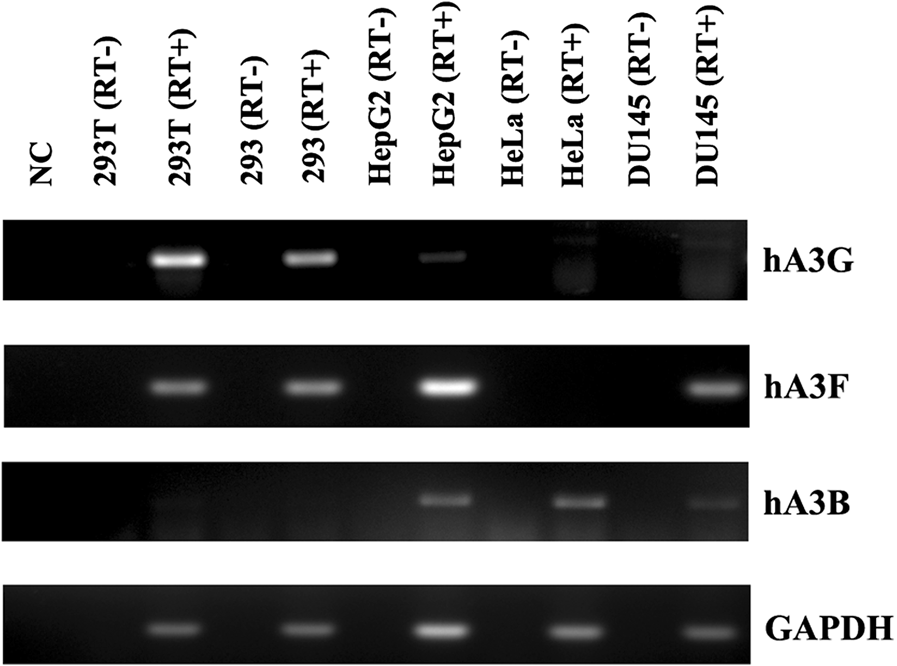
**RNA expression of APOBEC3s in different cell lines.** The cellular RNAs isolated from several human cell lines were subjected to RT-PCR with primers for human APOBEC 3 G (hA3G), hA3B, hA3F and GAPDH. The amplified PCR products were separated by agarose gels and visualized by ethidium bromide staining. (RT-), omission of reverse transcriptase from the reactions. NC, control with no added RNA.

Since glyco-gag counteracts the effects of mouse APOBEC3 [19], we considered the possibility that the glyco-gag sensitive restriction in HeLa cells might be due to one of the 7 human APOBEC3s. hA3G has been shown to potently suppress XMRV replication [28-31], while hA3B [30,31] and hA3F [29] have also been reported to have suppressive activity. The levels of hA3G, hA3B and hA3F in the different human cell lines were assessed by RT-PCR (Figure 6). hA3G transcripts were detectable in 293 and 293 T cells, even though they are generally considered to be functionally hA3G null. HeLa cells were of greatest interest, since they showed the highest levels of restriction for XMRV relative to MXMRV; in these cells only hA3B expression was readily detectable (Figure 6). Thus if the relative restriction of XMRV in HeLa cells results from an hA3 proteins, it would likely be hA3B. DU145 cells, which also showed relative XMRV restriction showed lower but detectable levels of hA3B transcripts. On the other hand, HepG2 cells that show relatively low levels of XMRV restriction also expressed hA3B. 293 and 293 T cells did not express appreciable hA3B (Figure 6). (While 293 and 293 T are functionally negative for hA3G, they in fact showed detectable transcripts by RT-PCR.)

APOBEC3 proteins are incorporated into HIV-1, mouse mammary tumor virus and MuLV virions, and they can inhibit viral replication in recipient cells [19,28,31-38]. MuLV and HIV-1 infection can also be restricted by APOBEC3 proteins in recipient cells [32,33], and glyco-gag can counteract mA3 restriction [19]. In light of the results of Figure 6, we tested if expression of hA3B in recipient 293 cells could restrict XMRV infection. 293 cells stably transfected with an epitope-tagged hA3B expression plasmid were used for titration of MXMRV and XMRV, and the resulting titers were compared to titration on 293 cells stably transfected with the backbone vector (pcDNA3) (Table 4). MXMRV had higher titers than XMRV on 293/pcDNA3 cells as expected, and the same was true in 293/hA3B cells. It was noteworthy that XMRV titers were indistinguishable on the 293/pcDNA3 and 293/hA3B cells. Thus hA3B did not restrict XMRV infection when expressed in the recipient cells. We also tested if expression of hA3B in virions affected infectivity of either XMRV or MXMRV. 293 T cells stably infected with these two viruses were transfected with the hA3B expression plasmid or pcDNA3, and the resulting viruses were titered on HeLa cells (Table 5). Expression of hA3B in the virus-producing cells did not affect the infectivities of either virus. Thus hA3B did not restrict these viruses when expressed in the donor infected cell. The lack of hA3B effect on XMRV infectivity was consistent with a previous report by Stieler et al.. [28]; in contrast two other groups have reported that XMRV is restricted by hA3B [29,30]. We confirmed that our hA3B expression vector resulted in production and packaging of the protein into XMRV virions (Additional file 3 Figure S3). The antiviral ability of our hA3B construct was confirmed by using it to incorporate hA3B into HIV-1 vector particles encoding GFP (pLVTHM, http://www.addgene.org/12247); the HIV-1 pseudovirus containing hA3B showed approximately 10-fold reduction of GFP expression in the recipient 293 T cells (Additional file 4 Figure S4), consistent with other reports [38,39]. These results indicated that the glyco-gag dependent restriction for XMRV in human cells appears to be APOBEC3-independent.

**Table 4:** Expression of hA3B in 293 cells did not alter infectivity of XMRV or MX viruses

**Virus**	**Recipient cells**	**Trial 1**	**Trial 2**	**Trial 3**	**Trial 4**	**Ave ± Std**	
		IU/ml^*^	**IU/ml**	**IU/ml**	**IU/ml**	**IU/ml**	Relative infectivity^**^
XMRV	293/pcDNA3	2.5 X 10^6^	2.9 X 10^6^	1.7 X 10^6^	2.9 X 10^6^	2.5 ± 0.52 X 10^6^	1 ± 0
	293/hA3B	2.2 X 10^6^	2.3 X 10^6^	2.5 X 10^6^	4.3 X 10^6^	2.8 ± 0.97 X 10^6^	1.17 ± 0.365
MX	293/pcDNA3	6.5 X 10^6^	6.9 X 10^6^	5.1 X 10^6^	8.3 X 10^6^	6.7 ± 1.3 X 10^6^	2.73 ± 0.231
	293/hA3B	6.9 X 10^6^	9.0 X 10^6^	3.6 X 10^6^	8.2 X 10^6^	6.9 ± 2.4 X 10^6^	2.72 ± 0.462

The 293 cells transfected with the plasmid encoding HA-tagged hA3B plasmids (293/hA3B) or control plasmids (293/pcDNA3) were selected by G418 and were used as recipient cells for infectivity assay. The recipient cells were infected with XMRV or MX viruses that were produced from the infected 293 T cells. *The infectious units (IU) were measured from the number of infectious centers that was normalized by the amount of Capsid in viruses and IU per ml was shown. **The relative infectivity to the 293/pcDNA3 infected with XMRV viruses was shown.

**Table 5:** Expression of hA3B in 293 T cells infected with XMRV/MX did not affect viral infectivity

**Donor cells**	**Trial 1**	**Trial 2**	**Trial 3**	**Trial 4**	**Ave ± Std**	
	ICs/ml^*^	**ICs/ml**	**ICs/ml**	**ICs/ml**	**ICs/ml**	Relative infectivity^**^
293T/XMRV/pcDNA3	7.0 X 10^4^	l.3 X l0^5^	l.5 X 10^5^	1.5 X 10^5^	l.2 ± 0.37 X l0^5^	1 ± 0
293T/XMRV/hA3B	8.5 X 10^4^	l.0 X l0^5^	l.4 X l0^5^	l.2 X l0^5^	1.1 ± 0.23 X l0^5^	0.931 ± 0.185
293T/MX/pcDNA3	8.4 X 10^5^	1.4 X 10^6^	3.2 X 10^6^	3.3 X 10^6^	2.2 ± 1.2 X 10^6^	16.7 ± 5.91
293T/MX/hA3B	1.0 X 10^6^	l.9 X 10^6^	4.1 X 10^6^	3.3 X 10^6^	2.6 ± 1.4 X 10^6^	19.8 ± 6.22

The infected 293 T cells were transfected with the plasmid encoding HA-tagged hA3B plasmids (293 T/XMRV/hA3B and 239 T/MX/hA3B) or control plasmids (293 T/XMRV/pcDNA3 and 293 T/MX/pcDNA3). HeLa cells were infected with XMRV released from the viral producing cells described above. *The infectious units (IU) were measured from the number of infectious centers that was normalized by the amount of Capsid in viruses and IU per ml was shown. **The relative infectivity to the HeLa cells infected with the virus released from 293 T/XMRV/pcDNA cells was shown.

### Glyco-gag is maintained in some but not all endogenous MuLVs

Endogenous retroviruses (ERVs) result from infection by an exogenous retrovirus into germ cells, and such proviruses can become permanent parts of the genomes of the progeny of that host. Endogenous retroviruses therefore provide insight into the prehistoric viruses at the time they endogenized into the genome. Included among the endogenous gammaretroviruses of mice are MuLV subgroups classified as ecotropic, xenotropic, polytropic or modified polytropic based on the nature of their envelope proteins (reviewed in [22]). The corresponding ERVs are referred to as Emv’s, *Xmv*’s, *Pmv*’s and *Mpmv*’s respectively. Laboratory mice represent relatively recent crosses between European mice (*Mus musculus domesticus*) and Japanese mice (*Mus musculus molossinus*) [40]. The endogenous MuLVs from *M. domesticus* are predominantly *Pmv*’s and *Mpmv’s*, while those from *M. molossinus* are predominantly *Xmv*’s [22,41]. It has recently been shown that XMRV is likely derived by recombination between 2 proviruses, and the endogenous *Pmv/Mpmv* giving rise to XMRV (preXMRV-2) [26] does not encode a functional glyco-gag. PreXMRV-2 is present as a single copy in the genomes of many (e.g. Harlan *nu/nu* mice) but not all mouse strains and has been identified in a few *M. m. domesticus* mice [26,42]. Thus at the time of endogenization, the then-exogenous preXMRV-2 likely did not encode glyco-gag.

**Figure 7:**
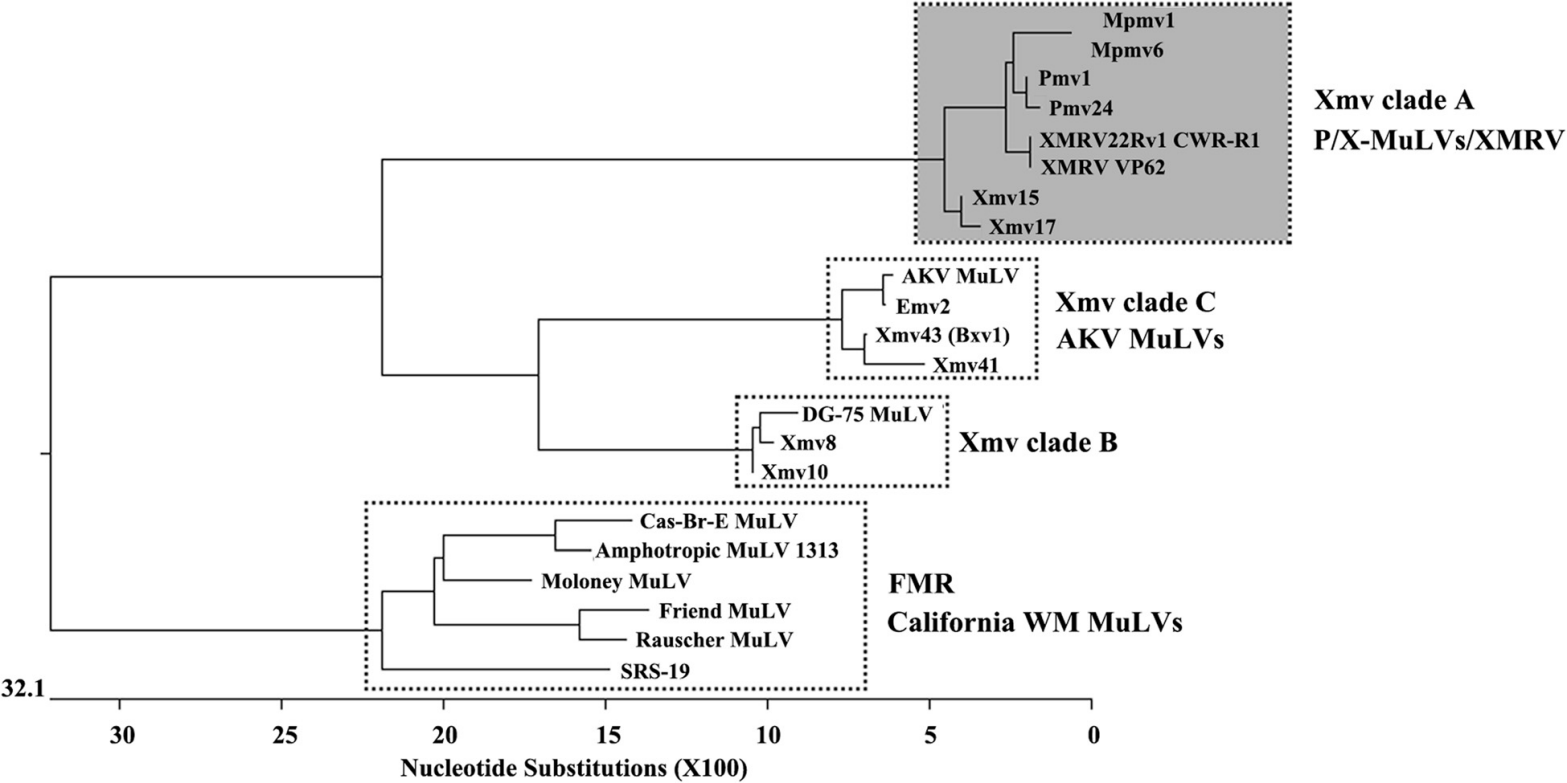
**Phylogenetic analysis of glyco-gag nucleotide sequences in MuLVs.** The nucleotide sequences in the glyco-gag region for endogenous MuLVs (nt 1–264, for M-MuLV J02255 nt 357 – 620) were aligned by DNASTAR Lasergene 8 MegAlign (Clustal W method) and a phylogenetic tree is shown. The same regions from representative exogenous MuLVs of the Friend-Moloney-Rauscher (FMR) group are also aligned. Box with the dark shading indicates endogenous MuLVs that do not have sequences capable of encoding glyco-gag. Boxes without shading contain viruses or endogenous MuLVs that can encode glyco-gag.

**Figure 8:**
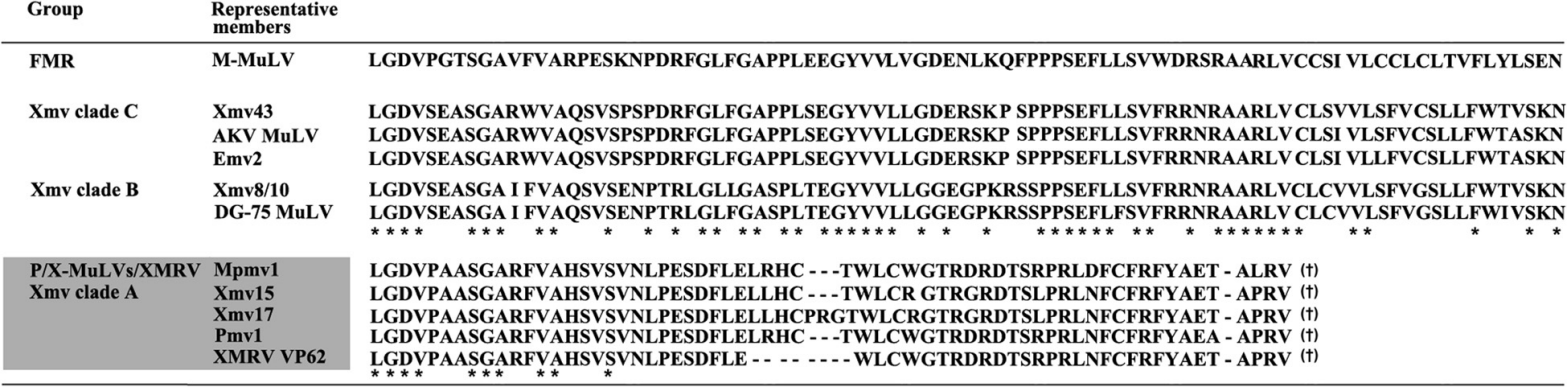
**Comparison of MuLV glyco-gag amino acid sequences.** Amino acid sequence alignments are shown for a representative FMR group virus (M-MuLV) and Xeno Clade B and C endogenous MuLVs at the top of the figure. For all of these cases, the glyco-gag reading frame is contiguous with the frame for Gag polyprotein. For Xeno Clade B and C, alignments are also shown for replication-competent viruses with high homology in the glyco-gag region (DG-75 MuLV and AKV-MuLV/Emv-2 respectively). *, amino acids shared among all of the glyco-gags. In the lower part of the figure (shaded box), predicted amino acids from endogenous MuLVs that cannot encode full-length glyco-gag are shown. The predicted open reading frames close before the beginning of the coding sequences for Gag polyprotein. *, predicted amino acids shared between these MuLVs and the glyco-gag positive MuLVs and MuLVs. The glyco-gag amino acid sequences in MuLVs were aligned by DNASTAR Lasergene 8 MegAlign (Clustal W method).

We investigated if the other endogenous MuLVs of mice had the capacity to encode a glyco-gag. The genome has been sequenced for C57BL6 (B6) mice. A bioinformatic search of the B6 genome identified 48 MuLVs that were full length or near full length: 1 endogenous *Emv*, 10 *Xmvs*, 23 *Pmvs* and 13 *Mpmvs*. The *Xmvs* were previously further subdivided into three clades (A, B and C) [43]. Phylogenies based on the nucleotide similarities in the glyco-gag regions (nt 1–264, for M-MuLV J02255 nt 357–620) are shown for representative proviruses in Figure 7, and predicted peptides beginning with the putative CUG initiation codon for glyco-gag are shown in Figure 8. Included in this analysis are specific exogenous MuLVs (e.g. AKV, DG75, and Moloney MuLVs). *Xmv* clade A proviruses grouped with *Pmv* and *Mpmv* proviruses, and also with XMRV/PreXMRV-2 (Figure 7). All of the endogenous viruses in this group could not encode a glyco-gag (Figure 8). Thus the primordial MuLVs in this group presumably could replicate in mice without glyco-gag, although none of these ERVs are known to produce infectious MuLVs.

In contrast, clade B and C endogenous xenotropic proviruses could theoretically encode classical glyco-gags, i.e. they contained a contiguous upstream coding sequence in the same reading frame as Pr65^*gag*^, beginning with a CUG codon. Moreover, the predicted amino acid sequences showed substantial amino acid identity with the consensus sequence for the current exogenous MuLVs (Figure 8). It is also interesting that Clade C glyco-gag is identical over the first 70 amino acids with the glyco-gag of two endogenous ecotropic MuLVs (AKV of AKR mice and *Emv2* of C57BL mice). Both AKV and *Emv2* can spontaneously activate and establish infections (and leukemias) in mice [44], although *Emv2* produces virus at much lower efficiency due to a single replacement mutation in *pol*[45]. The exogenous SL3-3 MuLV derived from AKV MuLV has an identical glyco-gag to that of AKV MuLV. Likewise the Clade B xenotropic proviruses *Xmv*-8/10 have a glyco-gag that is highly related (97.7% amino acid identity) to a replication-competent xenotropic MuLV originally found in released from human DG-75 leukemia cells [46]. In summary, two of the three clades of endogenous xenotropic MuLV proviruses can encode a glyco-gag, while one (Clade A) cannot. The endogenous *Pmv* and *Mpmv* proviruses cannot encode glyco-gag.

## Discussion

In this report, we used XMRV to investigate MuLV glyco-gag. XMRV is a recombinant between two endogenous proviruses in the mouse genome, preXMRV-1 and preXMRV-2, and it provides insight into the progenitor viruses that endogenized the mouse genome during evolution. The 5’ end of the XMRV genome, which is where glyco-gag is encoded, is derived from preXMRV-2. While XMRV does not encode a classical glyco-gag, there are two open reading frames in the 5’ end of the genome that could potentially encode glyco-gag activity. However, mutation of either of these potential reading frames had no measurable effect on viral release or infectivity, indicating that XMRV does not encode glyco-gag function. On the other hand, XMRV can respond to glyco-gag (albeit less than M-MuLV), since a chimeric XMRV encoding in-frame glyco-gag from M-MuLV (MXMRV) showed enhanced virus release through lipid rafts and enhanced infectivity. Expression of M-MuLV glyco-gag in trans could also enhance viral release and infectivity. Studies on the infectivities of MXMRV vs XMRV showed different effects in different human cell lines, which revealed relative restriction of XMRV in some human cells (e.g. HeLa) compared to others (e.g. 293); the restriction could be overcome by M-MuLV glyco-gag.

As shown in Figure 2, mutations designed to interfere with translation of two upstream ORFs in XMRV did not significantly affect infectivity or virus release, which allowed us to conclude that the putative 53 or 58 amino acid proteins were not important for viral replication. For one of these mutants, VP62Δgg1, there was approximately 80% reduction in infectivity. However, there was also an equivalent reduction in incorporation of viral RNA into released virus, which suggested that the reduced RNA incorporation was the cause of the reduced infectivity. For M-MuLV, there are several stem-loop structures in the region encoding glyco-gag (including the CUG initiation codon in one loop), and this region of the genome contains the RNA packaging (Psi) sequences [47]. It is thus likely that the analogous region of XMRV is important for RNA incorporation. Use of the M-fold program [48,49] on nucleotides 1–607 of XMRV predicted several stable stem-loops, and the nucleotide (382) mutated in VP62Δgg1 would normally be in a stem; the predicted structure of the mutant RNA showed an additional loop in this stem (T. Nitta and H. Fan, unpublished). The secondary structure of 5’ end of the XMRV genome predicted by SHAPE (selective 2’-hydroxyl acylation analyzed by primer extension) also suggested that the region corresponding to the mutation in VP62Δgg1 may form a stem structure, and that it may be involved in the formation of intermolecular loop-loop kissing interactions in RNA dimers [50]. This supports the idea that the region encompassing the VP62Δgg1 mutation is important in packaging of viral RNA into virions.

Reports from us and others have suggested there are multiple functions for glyco-gag, although the regions of glyco-gag responsible for the different activities remain to be fully elucidated [15-19]. In our studies describing enhancement of virus release through lipid rafts for MuLV and HIV-1, the unique N-terminal 88 amino acids (expressed by pHA-gg88) were sufficient for this activity [17]. Likewise, in this study, pHA-gg88 was sufficient to maximally enhance XMRV release from cells (Figure 3). On the other hand, additional glyco-gag sequences are required to enhance infectivity of *nef*-negative HIV-1 and glyco-gag negative MuLV from lymphocytes: the N-terminal 189 amino acids are required for full enhancement, and a 96 amino acid N-terminal fragment showed 60% activity [18]. Consistent with this, we found that HA-gg88 enhanced XMRV infectivity 40-60% as well as full-length gPr80^*gag*^ (Figure 5).

Studies on the infectivities of MXMRV vs XMRV in different human cells indicated that some cell lines (e.g. HeLa) showed substantially higher susceptibility/infectibility for MXMRV than for XMRV (≥ 20-fold, Table 3), while for other cell lines, the two viruses had more similar titers (≤ 5-fold difference); this reflected more efficient replication of XMRV in the latter cells. Glyco-gag in virions might affect viral factors (e.g. efficient incorporation of Env into virions or change of viral core nature), which would provide a missing activity (e.g. viral attachment or entry) required to infect certain cell lines. The results of infectivities would also be consistent with restrictive cells (e.g. HeLa) expressing a factor that restricts XMRV replication, but which can be overcome by glyco-gag. Cells such as 293 would not express or express lower amounts of the factor. Comparisons between cell lines that differ in relative restriction of HIV-1 have led to the discovery of cellular restriction factors for HIV-1 replication (such as APOBEC3G and Trim5alpha [51,52]), and our results might lead to identification of a novel restriction factor for XMRV in human cells. The experiments of Figure 6 and Tables 4 and 5 further indicated that the cell specific variation in restriction of XMRV observed here is not due to hA3 proteins, even though hA3G, and potentially hA3B and hA3F can restrict XMRV in vitro.

The exogenous MuLV that endogenized to form preXMRV-2 presumably could replicate in mice at the time of endogenization. Indeed the preXMRV-2 provirus contains full *gag, pol* and *env* genes, although it cannot encode functional glyco-gag. It was therefore of interest to investigate whether other endogenous proviruses in the mouse genome could encode glyco-gag or not. Endogenous MuLV proviruses have been grouped into ecotropic, xenotropic, and polytropic subgroups based on sequence homologies and whether their predicted Env proteins can mediate infection of murine and non-murine cells [22] (Additional file 5 Table S1). The polytropic ERVs have been subdivided into *Pmvs* and *Mpmvs*[53], and xenotropic ERVs (*Xmvs*) have been further subdivided into three clades (A, B and C) based on nucleotide sequence comparisons [43]. As shown and described in Figure 7, based on the 5’ (glyco-gag) regions, XMRV and preXMRV-2 grouped with clade A *Xmvs* and also the *Pmvs* and *Mpmvs*. None of these viruses could encode glyco-gag, indicating that the exogenous progenitors for all of these viruses could replicate in mice in the absence of glyco-gag. On the other hand, clade B and C *Xmvs* would be predicted to encode glyco-gags (Figure 7). It was interesting that replication-competent MuLVs with glyco-gags highly similar or identical to those of clade B and C xenotropic proviruses have been isolated – i.e. the leukemogenic SL3-3 MuLV (derived from the endogenous ecotropic MuLV AKV) for clade C and the xenotropic MuLV DG-75 for clade B [46]. Thus the exogenous progenitors for the clade B and C xenotropic proviruses encoded glyco-gag at the time of endogenization.

The results of Figure 7 raise the question as to when glyco-gag developed during evolution of the murine gammaretroviruses. At first glance the shape of the phylogenetic tree in Figure 7 might suggest that glyco-gag was present in more ancient gammaretroviruses, and that it was subsequently lost in the progenitors for clade A *XmvsPmvs*, and *Mpmvs*. Laboratory mice are hybrids between European mice (rich in *Pmv* and *Mpmv* proviruses) and Asian mice (rich in *Xmvs*) [40,54], and the cluster of ERVs lacking glyco-gag largely derived from the western European *M. m. domesticus*. Arguments exist for whether glyco-gag negative MuLVs preceded glyco-gag positive viruses, or vice versa. A glyco-gag – like protein (upstream ORF in-frame with the equivalent of Pr65^*gag*^ ) is encoded by gammaretroviruses of several other mammalian species (feline leukemia virus [2], Gibbon ape leukemia virus [M. Pizzato, personal communication] and koala retrovirus [55]). Moreover the putative glyco-gag coding sequence of FeLV is also present in two endogenous FeLV-related proviruses in the cat genome (T. Nitta and H. Fan, unpublished), so glyco-gag was present in the primordial FeLV genome at the time it endogenized in cats. This conservation would support development of glyco-gag before radiation of gammaretroviruses to these different species, although we have not tested if these putative glyco-gags have equivalent function.

On the other hand, if glyco-gag negative MuLVs developed from glyco-gag positive viruses, what would be the driving force for loss of glyco-gag? One possibility could be that mice developed a restriction factor against glyco-gag, so that loss of glyco-gag could have facilitated MuLV replication in these mice. Nevertheless, exogenous MuLVs (glyco-gag positive) replicate efficiently in laboratory mice suggesting they carry no such factor, and inactivation of glyco-gag reduces infectivity in vivo [19]. It was interesting that the 5’ region of the glyco-gag negative proviruses (and XMRV) have CUG codons and immediately downstream sequences that would encode amino terminal highly conserved glyco-gag amino acids (Figure 8). This would be more consistent with loss of glyco-gag from initially glyco-gag positive viruses.

## Conclusion

A functional glyco-gag is not encoded by XMRV. M-MuLV glyco-gag can compensate for this defect and enhance XMRV replication. The ability of glyco-gag to increase XMRV infectivity varies in different human cell lines, implying that there is a factor restricting XMRV replication in human cells and it can be overcome by glyco-gag. The ability of M-MuLV glyco-gag for enhancing XMRV replication is independent of hAPOBEC3. Comparative analysis with leader sequences in endogenous gammaretroviruses of mice indicated that the viruses that gave rise to some but not all clades of xenotropic *Xmvs* had the potential to encode glyco-gag.

## Methods

### Cells

Human cell lines 293 (kidney), 293T (kidney), HepG2 (hepatocyte carcinoma), DU145 (prostate carcinoma) and HeLa (cervical cancer) cells and rat NRK (kidney) cells were grown in Dulbecco’s modified Eagle’s medium supplemented with penicillin (100 U/ml), streptomycin (100 mg/ml) and 10% fetal bovine serum. For examining the relation between hA3B and glyco-gag, 293 cells were transfected with HA-hA3B and were selected by G-418 (Gold Biotechnology). The selected 293 cells were grown in Dulbecco’s modified Eagle’s medium supplemented with penicillin, streptomycin, 10% fetal bovine serum and 400 ug/ml of G-418.

### DNA constructs

The full-length XMRV molecular clone pVP62 was described previously [20]. The XMRV mutants containing stop codons in or adjacent to the putative glyco-gag, gg53 and gg58 were made by site-directed mutagenesis (Figure 1 and Table 1) according to standard techniques. Details will be provided upon request. pMXMRV was constructed as follows. First, the M-MuLV R, U5 and leader sequence region was PCR amplified from the M-MuLV plasmid p63-2 [56] using the primers 5’ - aaaaagcggccgcgcgccagtcctccgattgactg and 5’ – aaaaactcgagattctcagacaaatacagaaacac, and then cloned into pCR4-TOPO. The M-MuLV sequences from this plasmid were excised by digestion with XhoI and BsiWI and ligated into a subclone of the XMRV gag sequence (from pVP62) similarly digested with XhoI and BsiWI, to give pMX-TOPO. This placed the M-MuLV glyco-gag coding sequence in frame with the first amino acid of XMRV gag. The M-MuLV R, U5 and leader sequence regions as well as a portion of XMRV gag was isolated from pMX-TOPO by digestion with NotI and BsiWI, and cloned in place of the XMRV R, U5, leader peptide and gag sequence in pVP62 digested with the same enzymes to give pMXMRV. To abolish glyco-gag expression from MXMRV, the pMXMRV mutants, pMX-3 + 4 and pMX CTG/CA (Figure 4) were generated by site-directed mutagenesis. p8065-2 is an expression plasmid for M-MuLV glyco-gag, and has been described previously [15]. The M-MuLV gPr80^gag^ expression vectors, p8065-2-HBH and pEGFP-8065-2 S were generated from p8065-2 by standard cloning techniques. p8065-2-HBH expresses full-length chimeric glyco-gag, with the HBH-tag (two 6-his tags flanking a biotinylation site [57]) inserted in the BsrGI site in p15^MA^ region (M-MuLV sequence J02255 nt 921) of M-MuLV Gag. p8065-2S-GFP encodes enhanced jellyfish green fluorescent protein (EGFP) used to the N-terminus of glyco-gag; in this construct the AUG for Gag polyprotein (Pr65^*gag*^) was mutated (AUG → AUC) to reduce translation of Pr65^*gag*^ from this construct. pHA-gg88, which encodes only the N-terminal 88 amino acids of M-MuLV glyco-gag (with an N-terminal HA epitope) was described previously [17]. The APOBEC expression vectors, pcDNA3.1D.APOBEC3G.V5 (hA3G-V5) were described previously [58]. The plasmid encoding hA3B was obtained from OpenBiosystem. The hA3B sequence was amplified by the primers, 5’ - aaaaactcgagctcgag gccaccaatccacagatcagaaatccg and 5’ - aaaaagcggccgctcacgtgtgtgttctcctgaag. The amplified PCR product was digested with XhoI and NotI and ligated into an expression plasmid for HA-tetherin [59] digested with the same enzymes.

### Antibodies and Chemicals

Rabbit polyclonal anti-MuLV p30^CA^ antiserum was described previously [60]. For detection of hA3G-V5, mouse anti-V5 antibody was used (Invitrogen). We used anti-mouse IgG conjugated with horseradish peroxidase (Thermo Scientific) and anti-rabbit IgG conjugated with horseradish peroxidase (GE Healthcare) for Western blots. In the focal immunofluorescence assay, the goat anti-rabbit IgG conjugated with Alexa Fluor 488 (Invitrogen) was used to visualize foci of the infected cells. The Calphos Mammalian Transfection Kit (Clontech) and BioT (Bioland Scientific LLC) were used for transfections.

### Infectivity assay

Titrations of viruses (XMRV, MXMRV and their derivatives) were performed by a focal immunofluorescence assay described previously [15] with slight modification. Briefly, 2 x 10^5^ target cells were seeded per 6-cm plate 18–24 h prior to infection. Dilutions of the viral stocks were then adsorbed to the cells in the presence of 8 μg/ml polybrene (1,5-dimethyl-1,5-diazaundecamethylene polymethobromide, Sigma) for 16 hr, followed by the addition of growth medium. At 5 days post-infection, the cells were fixed by 4% paraformaldehyde and then were incubated with the blocking buffer containing 10% calf serum and 1% Triton X-100 in PBS. The cells were treated with anti-MuLV p30^CA^ for 2 h at 4 °C, washed with phosphate-buffered saline (PBS) supplemented with 1% bovine serum albumin, and then stained for 1 h at 4 °C with a secondary fluoresence-conjugated anti-rabbit IgG antibody. The plates were washed three times, and foci of immunofluorescence were counted with a UV microscope. The amount of Gag in virions was determined by Western blots and immuno-densitometry, and used for normalization of infectivity.

### Detection of viruses by western blots

Detection of viral antigens by Western blots and assessment of virus release was described previously [16]. In brief, the cells transfected with XMRV expressing plasimids or the infected cells were seeded on 6-cm dishes one day before measuring viral release. The media were replaced once and cells were incubated for 6 hours; and then both cells and viruses were gathered. The media were clarified by low-speed centrifugation, were passed through a 0.45 μm filter and viral particles, and were pelleted in a Beckman SW41 rotor at 77,000 *g* for 1 hour. The pelleted viral particles and corresponding cell lysates were analysed by SDS-PAGE and Western blots using anti-MuLV p30^CA^. To quantify viral release, each poly-Gag and CA bands in the cells and media was quantified with the densitometry software AlphaEaseFC (Alpha Innotech), and the percentage of released Gag divided by total Gag proteins (poly-Gag and CA) in cells and media was calculated. Different exposures of the blots were analyzed to ensure that densitometry was in the linear range.

### Detection of viral RNA in virions

The viruses were gathered from the 293 T cells transfected with the XMRV plasmids, pVP62 or pVP62Δgg0-4. The released viruses into media were harvested by centrifugation as described above. The RNA in the viral pellets was isolated by NucleoSpin RNAII (Macherey-Nagel) and treated with DNase (Deoxyribonuclease I, Amplification Grade, Invitrogen). Then, RNA was used for cDNA synthesis (qScript cDNA Synthesis Kit, Quanta Biosciences). The cDNA samples were subjected to real-time PCR with SYBR Green PCR Master Mix (Applied Biosystems) and primers for XMRV (forward primer 5’ – gtggcctacctgtccaaaaa and reverse primer 5’ – gggttgtttgaccagtgctt). The amount of CA in virions was determined by Western blots and immuno-densitometry and was used for normalization. The results are expressed at relative viral RNA levels compared to those released from the 293 T cells transfected with wild-type XMRV.

### Detection of APOBEC expression

Total RNA was isolated from cell lines with the RNA isolation kit, NucleoSpin RNAII. One μg of the RNA was treated with Deoxyribonuclease I, Amplification Grade (Invitrogen), and was used for synthesizing cDNA (qScript cDNA Synthesis Kit, Quanta Biosciences). The cDNA samples were subjected to PCR with the primers described previously [28]. GAPDH was also amplified as internal control.

### Glyco-gag sequences of endogenous MuLVs

Proviral sequences for the ecotropic, xenotropic and polytropic MuLVs were extracted from the sequenced C57BL genome using previously published information on their chromosomal locations, *env* subtype and coding potential [43,61] (Additional file 5: Table S1).

### Statistical analysis

A Student’s or Welch’s *t*-test with equal or unequal variances, respectively, was conducted to detect the difference between mean scores in the two groups, based on the equality test of two variances.

## Competing interests

The authors declare that they have no competing interests.

## Authors' contributions

TN designed the study, performed experiments, statistical analyses, interpreted the data and drafted the manuscript. SL, DH and MA performed experiments and provided helpful discussion regarding data analysis. CAK provided Glyco-gag sequences of endogenous MuLVs, interpreted the data, provided vigorous discussion and prepared the manuscript. HF supervised the project, designed the research, interpreted the data and prepared the manuscript. All authors read and approved the final manuscript.

## Supplementary Material

Additional file 1 **Figure S1. Enhancement of XMRV release by M-MuLV glyco-gag.** pVP62 (XMRV expression plasmid) and different M-MuLV glyco-gag expression vectors were co-transfected into 293T cells and 48 hr later Gag proteins in the cell lysates and the released viruses were detected by SDS-PAGE and Western blot with anti-p30^CA^ antibodies. Click here for file

Additional file 2 **Figure S2. Virus release by XMRV and MXMRV.** 293T cells were transiently transfected with pVP62 (XMRV expression plasmid) or pMXMRV (MX), and Gag proteins in the cell lysates and the released viruses were detected. Equivalent analysis was performed on DU145 cells productively infected with XMRV and MXMRV. Click here for file

Additional file 3 **Figure S3. Incorporation of hA3B into XMRV and MXMRV.** A) To assess the expression ability of the epitope-tagged hA3B expression plasmid, 293 cells were transfected with HA-hA3B and selected by G-418. hA3B protein in cell extracts from the selected 293 cells was detected by anti-HA antibodies. B) To generate XMRV and MXMRV (MX) viruses containing hA3B, 293T cells were transiently transfected with pVP62 or pMXMRV along with HA-hA3B or pcDNA3 (control). Gag and hA3B proteins in the cell lysates and the virions released from the transfected 293T cells were detected by SDS-PAGE and Western blotting for anti-p30^CA^ and anti-HA antibodies. hA3B was incorporated into XMRV and MXMRV virions with equivalent efficiency. Click here for file

Additional file 4 **Figure S4. Inhibition of HIV infection by hA3B.** 293T cells were transiently co-transfected with the expression vectors, pCMV-dR8.74 (HIV-1 Gag-Pol), pMD2.G (VSV-G), pLVTHM (HIV-1-based vector expressing EGFP, http://www.addgene.org/12247) along with HA-hA3B or pcDNA3 (control). A) HIV-1 Gag proteins and hA3B in the transfected 293T cells and viruses were detected by SDS-PAGE and Western blot with anti-p24 and anti-HA antibodies. The pseudoviruses with hA3B released from the transfected 293T cells were used for titering HIV-1 infectivity. B) The vector stocks produced in the panel A were used to infect fresh 293T cells, and the GFP-positive cells were counted 2 days post-infection. The relative numbers of GFP-positive cells, normalized for p24 in the vector stocks, are shown. The value for the control vector lacking hA3B (pcDNA3) was set at 1. C) As a second measure of infectivity, equal volumes of vector stocks shown in the panel A were used for infection of fresh 293T cells. At 3 days post-infection, the amount of EGFP protein in the infected cells was determined by SDS-PAGE and Western blotting with anti-EGFP antibodies. The Western blot data and the relative volumes of each sample loaded on the gel are shown. When corrected for the amount of vector (as assessed by p24 protein), the inhibition of HIV vector infection by HA-hA3B was consistent with the infectivity assay in the panel B. Click here for file

Additional file 5 **Table S1. Endogenous ecotropic, xenotropic, polytropic and modified polytropic MuLVs in the sequenced C57BL genome.** GenBank accession numbers are provided for the BAC clones containing the ERVs along with the positions of the viral sequences and the presence or absence of the consensus glyco-gag sequence is noted. Click here for file
