# Supplementary figures and images for: Moloney murine leukemia virus glyco-gag facilitates xenotropic murine leukemia virus-related virus replication through human APOBEC3-independent mechanisms
Source: Retrovirology. 2012 Jul 24;9:58. doi: 10.1186/1742-4690-9-58 (PMC3423011; doi:10.1186/1742-4690-9-58)

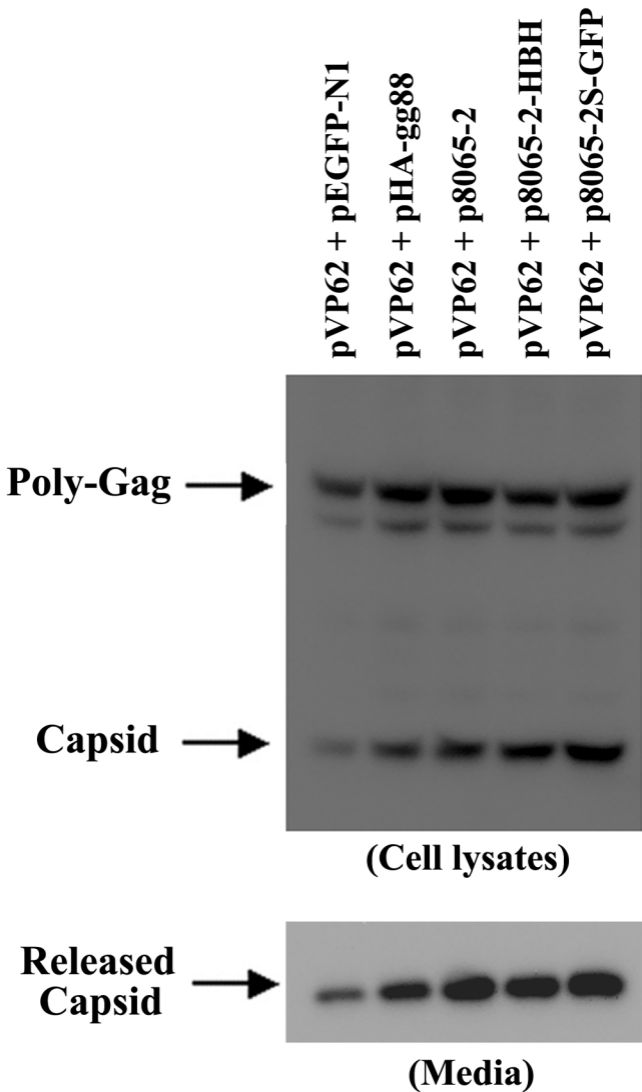

Supplement: Additional file 1 — Figure S1. Enhancement of XMRV release by M-MuLV glyco-gag. pVP62 (XMRV expression plasmid) and different M-MuLV glyco-gag expression vectors were co-transfected into 293T cells and 48 hr later Gag proteins in the cell lysates and the released viruses were detected by SDS-PAGE and Western blot with anti-p30CA antibodies. [file 1742-4690-9-58-S1.pdf]

**A**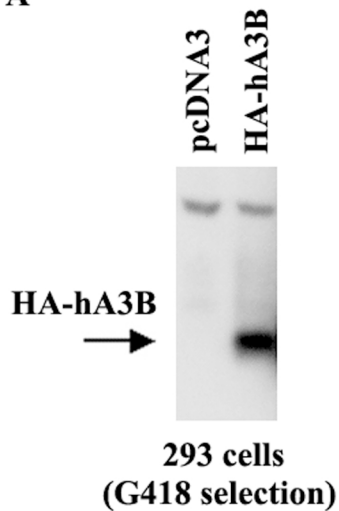**B**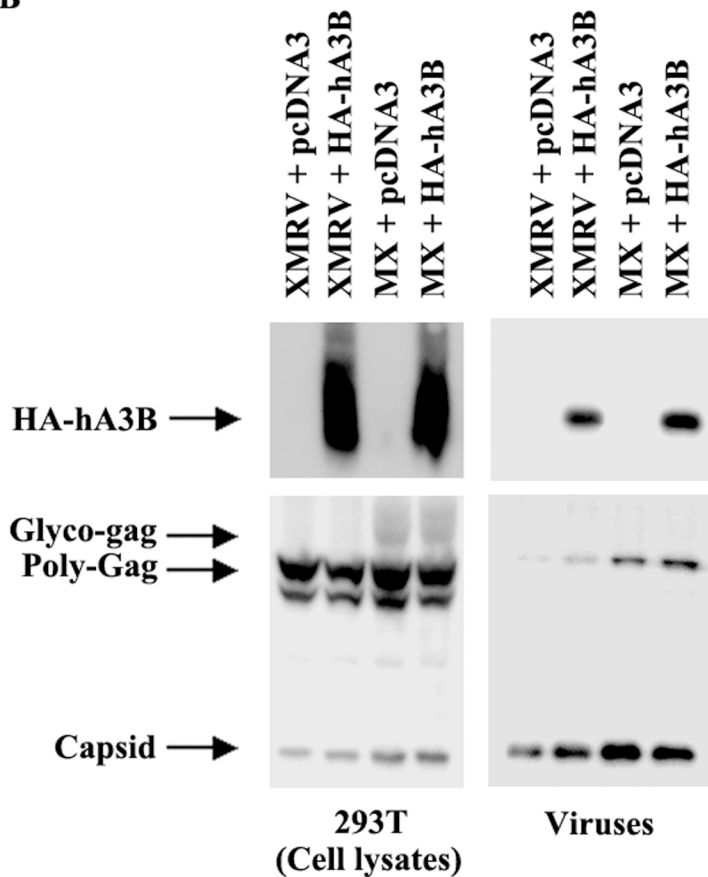

Supplement: Additional file 3 — Figure S3. Incorporation of hA3B into XMRV and MXMRV. A) To assess the expression ability of the epitope-tagged hA3B expression plasmid, 293 cells were transfected with HA-hA3B and selected by G-418. hA3B protein in cell extracts from the selected 293 cells was detected by anti-HA antibodies. B) To generate XMRV and MXMRV (MX) viruses containing hA3B, 293T cells were transiently transfected with pVP62 or pMXMRV along with HA-hA3B or pcDNA3 (control). Gag and hA3B proteins in the cell lysates and the virions released from the transfected 293T cells were detected by SDS-PAGE and Western blotting for anti-p30CA and anti-HA antibodies. hA3B was incorporated into XMRV and MXMRV virions with equivalent efficiency. [file 1742-4690-9-58-S3.pdf]

**A**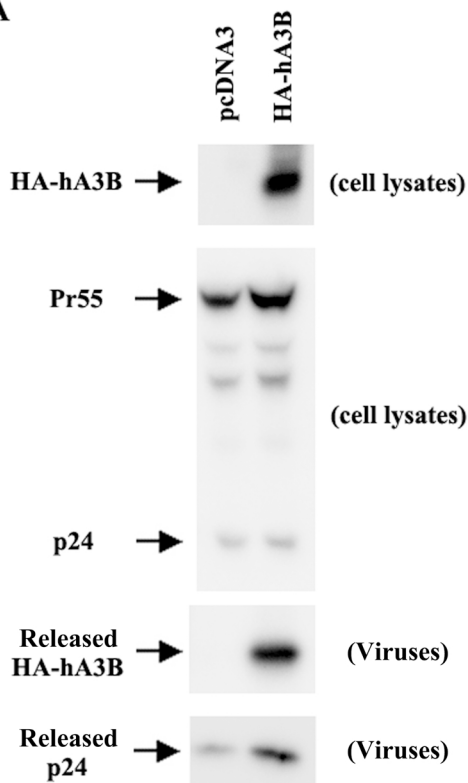**B**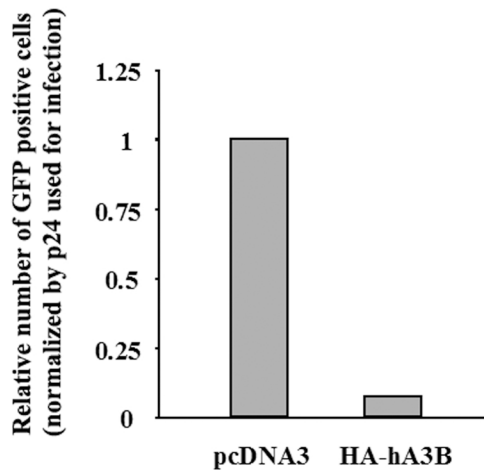**C**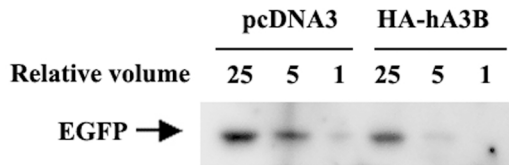

Supplement: Additional file 4 — Figure S4. Inhibition of HIV infection by hA3B. 293T cells were transiently co-transfected with the expression vectors, pCMV-dR8.74 (HIV-1 Gag-Pol), pMD2.G (VSV-G), pLVTHM (HIV-1-based vector expressing EGFP, http://www.addgene.org/12247) along with HA-hA3B or pcDNA3 (control). A) HIV-1 Gag proteins and hA3B in the transfected 293T cells and viruses were detected by SDS-PAGE and Western blot with anti-p24 and anti-HA antibodies. The pseudoviruses with hA3B released from the transfected 293T cells were used for titering HIV-1 infectivity. B) The vector stocks produced in the panel A were used to infect fresh 293T cells, and the GFP-positive cells were counted 2 days post-infection. The relative numbers of GFP-positive cells, normalized for p24 in the vector stocks, are shown. The value for the control vector lacking hA3B (pcDNA3) was set at 1. C) As a second measure of infectivity, equal volumes of vector stocks shown in the panel A were used for infection of fresh 293T cells. At 3 days post-infection, the amount of EGFP protein in the infected cells was determined by SDS-PAGE and Western blotting with anti-EGFP antibodies. The Western blot data and the relative volumes of each sample loaded on the gel are shown. When corrected for the amount of vector (as assessed by p24 protein), the inhibition of HIV vector infection by HA-hA3B was consistent with the infectivity assay in the panel B. [file 1742-4690-9-58-S4.pdf]
