# Supplementary material for: Moloney murine leukemia virus glyco-gag facilitates xenotropic murine leukemia virus-related virus replication through human APOBEC3-independent mechanisms
Source: Retrovirology. 2012 Jul 24;9:58. doi: 10.1186/1742-4690-9-58 (PMC3423011; doi:10.1186/1742-4690-9-58)
Supplement: Additional file 2 — Figure S2. Virus release by XMRV and MXMRV. 293T cells were transiently transfected with pVP62 (XMRV expression plasmid) or pMXMRV (MX), and Gag proteins in the cell lysates and the released viruses were detected. Equivalent analysis was performed on DU145 cells productively infected with XMRV and MXMRV. [file 1742-4690-9-58-S2.pdf]

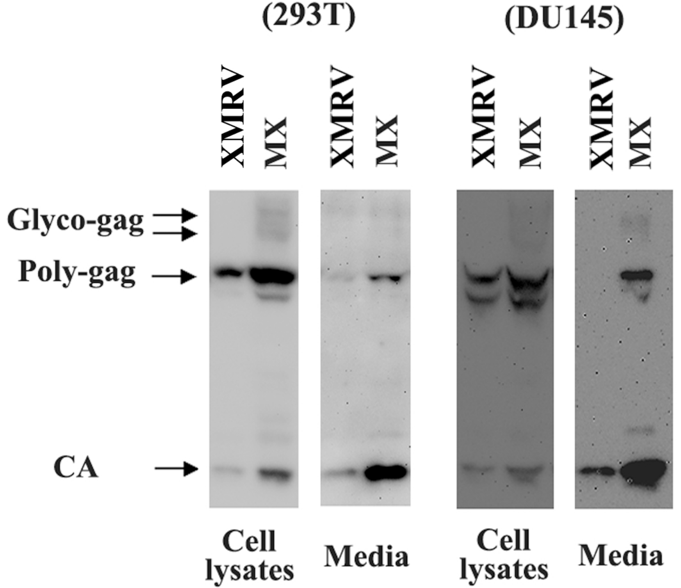
(293T)
(DU145)
XMRV
MX
XMRV
MX
XMRV
MX
XMRV
MX
Glyco-gag
Poly-gag
CA
Cell lysates
Media
Cell lysates
Media
